# From Trauma to Tumor: A Case of Progressive Gastrointestinal Stromal Tumor Mimicking Post-Traumatic Hematoma with Late-Stage Recurrence

**DOI:** 10.51894/001c.144538

**Published:** 2025-09-30

**Authors:** Aqsa Kanwal

**Affiliations:** 1 College of Osteopathic Medicine Michigan State University

## 92

### INTRODUCTION

Gastrointestinal stromal tumors (GISTs) are rare mesenchymal neoplasms often underdiagnosed due to their asymptomatic nature or incidental discovery. Though trauma does not cause GIST, it can unmask them. This case illustrates an abdominal injury initially mistaken for a hematoma, revealing a GIST that progressed to metastasis despite surgery.

### CASE DESCRIPTION

A 49-year-old male with a history of tobacco use presented with right-sided abdominal pain, bloating, and indigestion after falling from a truck. CT revealed an 8.3 x 8.6 x 7.2 cm heterogeneous mass near the small bowel with surrounding high-density fluid, concerning for a hematoma.

Months later, he returned with worsening periumbilical and right lower quadrant pain, decreased appetite, nausea, and chills. A repeat CT demonstrated mass enlargement to 13.1 x 10.7 x 12.4 cm, concerning for malignancy. Differential diagnoses included non-Hodgkin’s lymphoma, leiomyosarcoma, GIST, and neuroendocrine tumor.

A CT-guided biopsy confirmed GIST, characterized by spindle cells staining positive for vimentin, SMA, DOG-1, and CD117. He developed worsening abdominal pain, lightheadedness, chills, intermittent melena, and leukocytosis raising concern for superimposed infection. He was stabilized with antibiotics and successfully underwent exploratory laparotomy with en bloc resection of the abdominal mass, small bowel, and right colon.

Post resection in 2018, he did not adhere to regular oncology follow-ups. In February 2024, he developed abdominal symptoms, and GIST recurrence was suspected from incidental findings on an abdominal ultrasound for an inguinal hernia; CT findings also raised concern for recurrence. Metastasis initially made him a poor candidate for tumor resection. He was started on imatinib, leading to remission on repeat PET scan. In December 2024, he successfully underwent massive multidisciplinary cytoreductive debulking surgery of affected anatomical structures.

### DISCUSSION/CONCLUSIONS

Even in cases of classic abdominal trauma, malignancy should remain a consideration. Initially presumed to be a post-traumatic hematoma, progressive enlargement of the mass and worsening symptoms warranted further evaluation, ultimately leading to the diagnosis of GIST. This case highlights the need for consistent oncology and primary care follow-ups, along with risk factor counseling. Thorough evaluation of atypical presentations is crucial for timely diagnosis and optimal patient outcomes.

